# A birefringent spectral demultiplexer enables fast hyper-spectral imaging of protoporphyrin IX during neurosurgery

**DOI:** 10.1038/s42003-023-04701-9

**Published:** 2023-03-30

**Authors:** Mikael Marois, Jonathan D. Olson, Dennis J. Wirth, Jonathan T. Elliott, Xiaoyao Fan, Scott C. Davis, Keith D. Paulsen, David W. Roberts

**Affiliations:** 1 Thayer School of Engineering, Dartmouth College, Hanover, NH USA; 2 Geisel School of Medicine, Dartmouth College, Hanover, NH USA; 3 Dartmouth-Health, Dartmouth-Hitchcock Medical Center, Lebanon, NH USA

**Keywords:** Biological fluorescence, Surgical oncology

## Abstract

Hyperspectral imaging and spectral analysis quantifies fluorophore concentration during fluorescence-guided surgery^1–6^. However, acquisition of the multiple wavelengths required to implement these methods can be time-consuming and hinder surgical workflow. To this end, a snapshot hyperspectral imaging system capable of acquiring 64 channels of spectral data simultaneously was developed for rapid hyperspectral imaging during neurosurgery. The system uses a birefringent spectral demultiplexer to split incoming light and redirect wavelengths to different sections of a large format microscope sensor. Its configuration achieves high optical throughput, accepts unpolarized input light and exceeds channel count of prior image-replicating imaging spectrometers by 4-fold. Tissue-simulating phantoms consisting of serial dilutions of the fluorescent agent characterize system linearity and sensitivity, and comparisons to performance of a liquid crystal tunable filter based hyperspectral imaging device are favorable. The new instrument showed comparable, if not improved, sensitivity at low fluorophore concentrations; yet, acquired wide-field images at more than 70-fold increase in frame rate. Image data acquired in the operating room during human brain tumor resection confirm these findings. The new device is an important advance in achieving real-time quantitative imaging of fluorophore concentration for guiding surgery.

## Abstract (web summary)

A wide-field imaging system uses birefringence to efficiently permit quantitative fluorophore concentration mapping, with clear relevance for neurosurgery.

## Introduction

Image-guidance methods relying on pre-operative assessment of tumor location are limited by brain shift and other changes that occur during surgery^7^. Fluorescence-guided surgery (FGS), which does not depend on pre-operative image co-registration, is free of these limitations and is an effective way of identifying exposed tumor during a surgical procedure^4,6,8–11^. Fluorescence guided neurosurgery using 5-aminoluvelinic acid (ALA)-induced protoporphyrin IX (PpIX) is an FDA-approved procedure that leverages differential accumulation of the endogenous fluorophore PpIX in tumor cells after oral administration of ALA. The approach has been shown to improve surgical outcomes in the removal of high-grade gliomas^12,13^, as well as meningiomas and metastatic brain cancers^11,14–16^, and most commercial neurosurgical microscopes can be adapted with an ALA-PpIX module for surgical guidance.

Previous research has shown that quantification of PpIX concentration increases both sensitivity and specificity of tumor detection^1,3,5,14,17–22^, and is particularly valuable when visible fluorescence is not observed^3,5^. This functionality is critical, as post-operative patient survival is linked to completeness of tumor removal^23^. Optical fiber probes have been used to quantify PpIX fluorescence with high accuracy^3,14,17–20^. While results from probe measurements determine fluorescence intensity at a specific location, and quantify PpIX concentration in some cases, spatially locating the extent of disease and its associated boundaries with a probe is inefficient and impractical for neurosurgeons in the operating room (OR). To enable clinical adoption of quantitative fluorescence imaging in neurosurgery, widefield systems that rely on liquid crystal tunable filters (LCTF) to acquire images at multiple wavelengths have been developed to generate quantitative fluorophore concentration maps corresponding to the surgeon’s field-of-view^1,5,21,22^. Although these images provide valuable information on tumor location and extent of disease intraoperatively, acquisition times can be lengthy (>10 s) depending on the number of wavelengths being collected.

To reduce time delays and their impact on surgical workflow and to facilitate adoption of quantitative fluorescence imaging during neurosurgical procedures, an image replicating imaging spectrometer (IRIS) relying on birefringent spatial demultiplexing has been designed and developed to acquire full hyperspectral stacks of 64 channels of different wavelengths and polarizations simultaneously, leading to reduced acquisition times. In this paper, channel responses of IRIS were characterized, and the system was calibrated to generate quantitative PpIX concentration maps. A set of PpIX dilutions was measured with both IRIS and an earlier LCTF imaging system to compare accuracy of quantitative estimates generated over a wide range of PpIX concentrations and associated phantom optical properties. Both these systems were also deployed during a high-grade glioma resection procedure to image tumor and assess relative performances of the two instruments in the OR.

To the best of our knowledge, this works presents the highest number of image replication channels ever to be realized, at least to date (by 4-fold), and more importantly, the first application of the technology intraoperatively to achieve near-video rate (4–6 Hz) acquisition of wide-field images of the absolute concentration of PpIX – a fluorophore used widely in molecularly-guided neurosurgery. Here, we show head-to-head comparisons, including in the OR, of the new imaging system relative to our earlier (and much slower) LCTF technology. The IRIS system demonstrated improved linearity of response to PpIX concentration compared to the LCTF instrument and a factor of 70 (or more) increase in frame rate. While system-use described in this paper centers on acquisition of quantitative PpIX fluorescence images for surgical guidance, its broad wavelength range (~425 nm to ~825 nm) makes the technology suitable for a wide range of fluorescent agents and/or wideband hyperspectral reflectance applications.

## Results

### Optical channel response

**Fig. 1:**
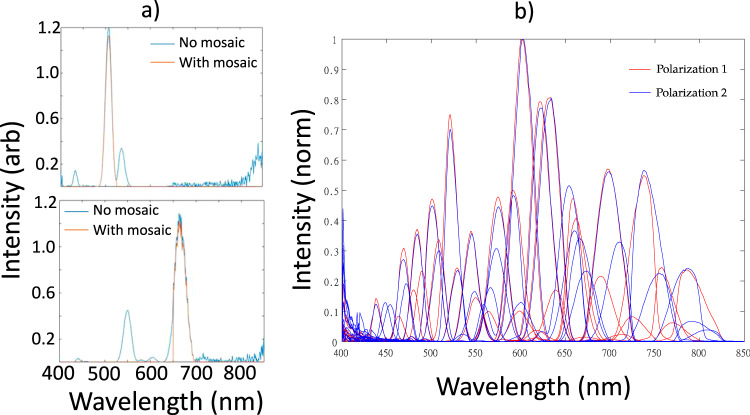
Spectral characteristics of IRIS channels. **a** Measured spectral response of IRIS in two representative channels with and without the mosaic filter. Side lobes from the BSD alone are present in the spectrum. Interference filter mosaic removes side lobes in each channel. **b** Simulated spectral response of all channels overlaid on one another.

Spectral characteristics of the IRIS channels are shown in Fig. 1. Data are split into two sets of 32 channels with orthogonal polarizations. Channel to channel transmission responses vary in intensity and bandwidths as expected. Wavelengths ranging from ~425 to ~825 nm are detected with peak transmission occurring for channels situated in the 575nm–650nm band. Because the transfer function of each channel was recovered using a tunable filter to scan through wavelengths, the transmission curves are coupled with the wavelength response of the LCTF which explains noise in the data in the low 400 nm (LCTF has poor efficiency there).

### IRIS Specific PpIX spectra

**Fig. 2:**
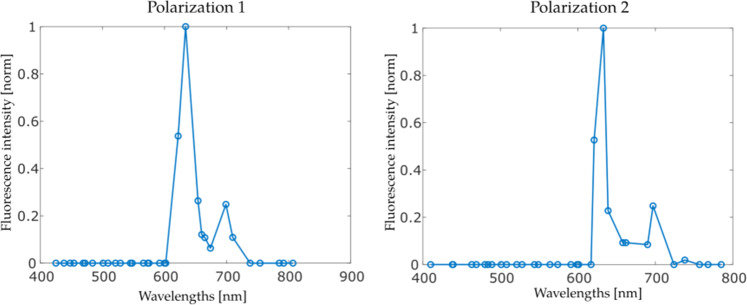
PpIX spectra acquired with IRIS. IRIS specific PpIX spectra for the two orthogonal polarizations acquired. Each marker represents data from one optical channel.

Normalized PpIX spectra acquired with IRIS, averaged over many optical properties and concentrations, are shown in Fig. 2. Here, combinations of blood volume fraction (BVL) and intralipid concentration (IL) in the phantoms were [1%IL, 2%BVF], [1.5%IL, 2%BVF], [2%IL, 2%BVF], [1.5%IL, 1%BV,], and [1.5%IL, 3%BVF] for a fixed PpIX concentration (1 µg/ml) similar to past experiments^5,24^. Differences in the spectral shape between polarizations can be attributed to the mosaic interference filter attenuating different passbands and sidelobes in each case.

### Sensitivity curves

**Fig. 3:**
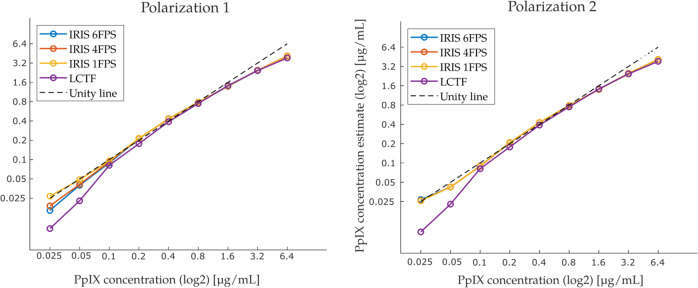
Imaged PpIX concentrations compared to actual PpIX concentrations in phantoms. Correlation between concentration estimates computed with LCTF and IRIS systems relative to actual phantom PpIX concentrations for both IRIS polarizations.

IRIS PpIX concentration estimates obtained after spectral fitting at various PpIX concentrations are shown in Fig. 3 as a function of actual PpIX concentration. These sensitivity curves include data collected with IRIS at exposure times of 0.167 s (6 FPS), 0.25 s (4 FPS), and 1 s (1 FPS), as well as with our existing LCTF system^5,20^ at 0.25 s per wavelength over 42 wavelengths. The LCTF instrument loses linearity at the lower PpIX concentrations while the IRIS system produced a more linear response at these levels (down to 0.025 µg/ml, the lowest PpIX concentration evaluated here). Both systems deviated from the unity line at high concentrations. Using these data, a scaling factor was computed to relate imaged concentration estimates to actual phantom concentration values, allowing concentration maps computed from IRIS data to be quantitative.

### Feasibility study in human glioma surgery

**Fig. 4:**
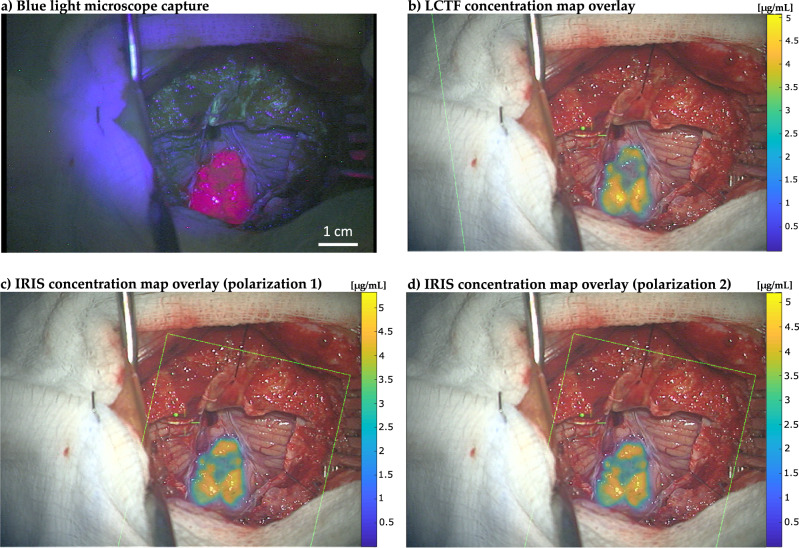
Comparison of visual PpIX fluorescence and quantitative concentration maps imaged during neurosurgery. **a** Visible fluorescence of PpIX illuminated under blue light. **b** PpIX concentration map generated from hyperspectral data acquired with the LCTF. **c** PpIX concentration map generated with IRIS using channels from the 1^st^ polarization. **d** PpIX concentration map generated with IRIS using channels from the 2^nd^ polarization. Concentration maps are overlaid on RGB images of the surgical field of view acquired with the Pentero operating microscope. Green box represents the field of view of the hyperspectral imaging system used to detect fluorescence. Color scale bars show imaged PpIX concentrations in micrograms per milliliter (μg/mL). Spatial scale bar (1 cm) appears in **a**).

PpIX concentration maps obtained in human brain tumor during resection with IRIS and LCTF systems are shown in Fig. 4. To display concentrations in areas of high importance preferentially, an opacity map proportional to concentration value was applied to the overlays. Differences in acquisition time between the two systems are substantial; the LCTF consumed about 12 s to scan through 42 wavelengths whereas the IRIS acquired 64 hyperspectral channels simultaneously in 167 ms, a factor of 71.9 faster. Examination of overlaid PpIX concentration maps suggests that values and their distribution recovered with IRIS match counterparts generated with LCTF. Concentration values from both systems are in the same range, with regions of highest fluorescence registering about 5 µg/mL, which is a common value for high-grade tumor fluorescence that is easily visible to the surgeon. The correlation coefficient between the LCTF and IRIS images, a measure of image similarity, was found to be 0.90, indicating a high degree of similarity. Finally, no difference was found between concentration maps generated by each IRIS polarization as expected since PpIX fluorescence emission from tissue is not known to be anisotropic.

## Discussion

The IRIS hyperspectral fluorescence imaging system described in this paper acquires 64 channels of varying wavelength and polarization responses simultaneously to form absolute PpIX concentration images coregistered with the surgeon’s field of view through an operating microscope at rates up to 6 frames per second (FPS). The new instrument exceeds channel counts of prior image replicating imaging spectrometers by four-fold^25^, and also differs from previous realizations by accepting unpolarized incoming light, which increases overall optical efficiency and provides additional information on polarization state of incoming light. Hyperspectral data acquired for both polarizations are treated independently, and concurrent fluorophore concentration maps are generated. While polarization sensitive images have not provided additional information in fluorescence-guided neurosurgery to date, other studies have shown that polarization sensitivity has applications in molecular interaction quantification^26–28^ and skin analyses^29–31^.

Liquid phantom experiments showed that our sister LCTF system underestimated concentrations lower than 0.1 µg/mL whereas IRIS estimates remained accurate for the full range of PpIX phantoms evaluated here (as low as 0.025 µg/ml). Thus, the threshold for accurate sensing of PpIX concentration appears to be 4 times lower with IRIS relative to its LCTF counterpart. The improvement is likely attributed to IRIS camera specifications being optimized for low light fluorescence applications, combined with the minimal amount of light rejection inherent to image replication techniques. Moreover, collecting light in a single image acquisition, rather than acquiring contributions in a series of filtered images, minimizes noise amplification and yields higher signal-to-noise ratio. Concentration estimates from both systems were underestimated for phantoms with values above 1.6 µg/mL, which could be due to PpIX aggregation inhibiting fluorescence at higher concentrations^32,33^.

Images acquired with IRIS during a surgical procedure confirmed its rapid frame rate which did not degrade resulting concentration maps relative to ones generated from multi-spectral data collected with a conventional LCTF. To the best of our knowledge, the results presented here represent the first time an image replicating system has been used in the fluorescence guided surgery context. The speed with which the system captures hyperspectral data (up to 6 FPS) provides rapid feedback in terms of concentration maps overlaid on the surgeon’s view. Time required to produce overlays is limited by image registration computations and other data transfer overhead arising from lack of direct access to camera data. The latency caused by both of these steps is readily remedied through software optimization, which is now underway, and will allow concentration maps to be updated multiple times per second. Spectral fitting capabilities of IRIS are currently focused on PpIX as the fluorophore for which IRIS specific spectra were generated. However, we intend to produce a spectral conversion matrix, allowing IRIS specific responses to be generated for any fluorophore with emissions in its spectral range. This accomplishment would enable more complex processing, such as white light correction from reflectance measurements^1,20,34^ to account for blood and tissue absorption when computing concentration estimates.

While ALA-PpIX guidance is approved for high grade glioma (HGG) in the US at this time, and the example shown in Fig. 4 was confirmed pathologically to be HGG, accurate fluorescence guidance for low grade glioma (LGG) surgery could have a major impact on this patient population. The literature on ALA-PpIX in LGG suggests heterogeneity in fluorescence emissions from these tumors^11–13,34,35^. However, data acquired with imaging systems similar to the IRIS device reported here (e.g., with its companion LCTF and related probe systems) in LGG under IND-regulated protocols indicate fluorescence detection is possible even though the emissions are not always visible to the surgeon^2,5,36–38^.

In summary, a wide-field imaging system for quantitative fluorophore concentration mapping using birefringence was designed, fabricated and tested to accelerate hyperspectral acquisition times during fluorescence-guided neurosurgery. The instrument is able to acquire the spectral information needed to compute quantitative concentration maps in a fraction of a second. PpIX concentration maps were acquired in human glioma surgery using both IRIS and LCTF systems, and the two units showed robust agreement. Near-video-rate hyperspectral image analysis and visualization in the microscope oculars should be achievable with improvements in image co-registration speed. Other applications taking advantages of the inherent separation of polarization of this system are under investigation as well.

## Methods

### IRIS Instrument

Central to the new imaging instrument is an Image Replicating Imaging Spectrometer (IRIS) conceptualized by Harvey et al. ^39^, which exploits birefringence to split incoming wavelengths onto different sections of a camera sensor. Having wavelengths separated spatially, rather than temporally scanning narrow transmission bands, increases acquisition throughput substantially and records a full hyperspectral cube in a single acquisition. Consequently, the approach is much faster than sequential wavelength filtering and mitigates the impact of temporal artifacts inherent to sequential data acquisition. The primary trade-off compared to systems that acquire spectral bands sequentially is a loss of spatial resolution which arises from the fact that IRIS spatially divides the light into spectral components, sending different wavelength bands to different regions of the image sensor. The impact of this compromise is specific to the application and depends on a number of factors, including: 1) the resolution of the optical system, 2) the required spatial resolution for the application, and 3) the importance of temporal resolution. This trade-off is discussed further below.

**Fig. 5:**
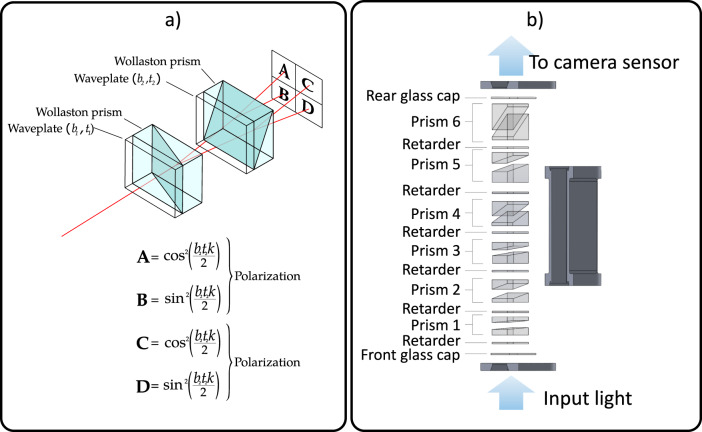
Birefringent spectral demultiplexing and IRIS birefringent spectral demultiplexer design. **a** Diagram of a four-way birefringent spectral demultiplexer. Incoming signal is split into four different regions which can be imaged with a camera sensor. **b** Design used for IRIS which produces two sets of 32 channels with orthogonal polarizations.

Spatial multiplexing is achieved through a birefringent spectral demultiplexer (BSD)—the optical component responsible for efficient redirection of wavelength bands to different sections of the camera sensor. BSD exploits birefringence filtering implemented through a succession of alternating waveplates and Wollaston prisms. Each waveplate-prism pair splits the incoming signal into two separate polarizations having transfer functions:

1 $${T}_{\parallel }\left(k\right)={\cos }^{2}$$

2 $${T}_{\perp }\left(k\right)={\sin }^{2}\left(\frac{{btk}}{2}\right)$$

where *b* is the birefringence of the waveplate, *t* is its thickness, and *k* is the wavenumber. The working principle of a 4-way birefringent spectral demultiplexer is illustrated in Fig. 5.

**Fig. 6:**
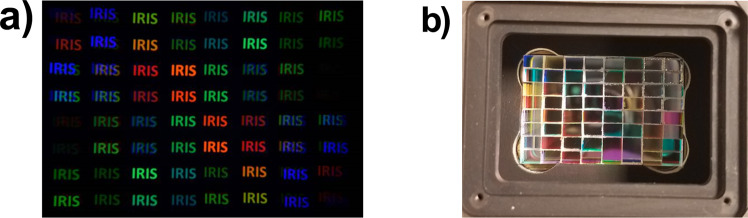
Birefringent spectral demultiplexer output and mosaic interference filter. **a** BSD view of the word “IRIS” displayed in white letters on a computer monitor. Channels that appear dark have their peak transmission wavelength falling outside of the emission range of the computer monitor. **b** Hand crafted mosaic interference filter mounted on the camera sensor.

This example uses two waveplate and Wollaston prisms; however, by combining N waveplate-prism pairs, an image is split into 2^N^ hyperspectral planes; the global transfer function for each plane is the product of the transfer functions that acted on the light rays reaching that plane. Previous implementations have been reported with up to sixteen channels^39–42^. Herein, we designed a six-prism system (Fig. 1b) which splits the incoming signal into 64 channels of different wavelengths and polarizations. A BSD typically polarizes incoming light linearly and ensures that the signal is separated into orthogonally polarized waves of equal amplitude. However, we left out the input polarizing filter to detect incoming polarization information, and to increase overall light sensitivity. With this arrangement, the first Wollaston prism in the system divides the incoming signal in two orthogonal polarizations that are split into two sets of 2^N-1^ channels with identical channel responses but different polarizations. To demonstrate the splitting capabilities of the system, a picture of the view through the demultiplexer pointing at a white target (the word “IRIS” displayed in white letters on a computer monitor) is shown in Fig. 6.

Optical design of IRIS required consideration of several parameters to ensure high light throughput, adequate spectral separation, and localization on the image sensor. Specifically, an optimal combination of waveplates were identified by varying waveplate thickness (*t*) and wavenumber (*k*) in the polarization rotation functions provided above over a wide range using MATLAB. Light transmission through the Wollaston prisms was simulated using the ZEMAX optical modeling software package. Additionally, filter transfer functions from the interference filter supplier were incorporated in the computations, which enabled selection of bandpass filters for the mosaic that maximized transmission in the bandpass while removing contaminating side lobes.

The mosaic filter was crafted from commercial glass interference filters that were cut, glued, and manually fitted directly onto the image sensor as shown in Fig. 6b. Since interference filters were only used to remove side lobes, and most of the light was redirected rather than filtered, high optical efficiency was achieved in the system design.

**Fig. 7:**
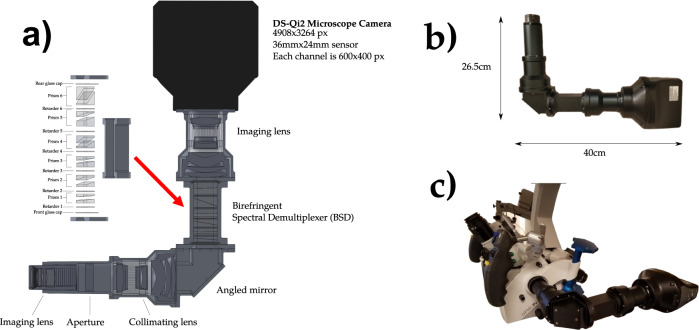
IRIS component diagram and system dimensions. **a** Internal components and optics modeled for the IRIS. **b** Final dimensions of the system. **c** IRIS mounted on the Pentero surgical microscope.

The remaining optical components, e.g. imaging lenses, aperture, collimating lens and mirror, were designed using manual lens calculations followed by confirmation using ZEMAX, which provides ray tracing for any optical system. These simulations were used to choose and position optical components that (1) maintain high light throughput, and (2) ensure that the light in each channel from the BSD was properly positioned on the image sensor and had appropriate scale. The imaging sensor chosen was a 16.25 megapixels DS-Qi2 microscope camera (Nikon Instruments Inc, Melville, NY), which was selected for its low read noise, high dynamic range, good linearity, and most importantly, the large 36 × 24 mm surface area of its CMOS sensor. Resulting images have a resolution of 4908 × 3264 pixels, with each channel dedicated to a 600 × 400 pixel region. A diagram of the internal schematics, as well as the final dimensions of the system, can be seen in Fig. 7.

### Basic optical characterization: spatial resolution and field of view

As described above, IRIS divided the imaging array into wavelength channels, and the number of pixels available per channel was limited compared to systems which sample wavelengths sequentially on a full sensor array. In the current implementation, each IRIS channel consists of 400 × 600 pixels, whereas the LCTF system makes use of a full 2048 × 2048 imaging sensor for each wavelength. However, in practice, the spatial resolution was limited by the optics of the surgical microscope on which both units were mounted when in use. Using a standard USAF resolution test chart, image resolution was found to be identical (0.63 mm) for IRIS and LCTF systems, and accordingly, they operated with similar spatial resolutions as implemented here. The IRIS system’s field of view at the typical 25 cm working distance for the surgical microscope was approximately 6 × 9 cm. Images from which spatial resolution was determined for the IRIS and LCTF systems scaled (windowed and leveled) for visualization are provided as Supplementary Figs. 1–2 (raw 16 bit image data can be found in Supplementary Data 3; also see Data Availability).

### Spectral responses of the channels

To corroborate channel responses that were simulated optically, channel bandwidth was recorded by imaging a 99% Spectralon® diffuse reflectance target (SRT-99; LabSphere) with a calibrated white light source (SL1 Tungsten-Halogen, StellarNet Inc., Tampa, FL) and placing an LCTF (Varispec, PerkinElmer, Waltham, MA) at the input of the optical system. In this configuration, the full spectrum white light source is incident upon the LCTF, which allows only a selected waveband to pass into the IRIS. By sequentially selecting wavebands over the wavelength range of interest (400–900 nm) in steps of 1 nm, and acquiring images with the IRIS at each waveband, the spectral response of the system was recorded. Because LCTF’s have a limited spectral operating range, we used a visible-spectrum LCTF to cover 400–700 nm and a near-infrared LCTF for 650–900 nm. These data were combined, and average values in a 50 pixel radius circle in the center of each channel image were analyzed to generate spectral sensitivity curves for all 64 channels.

### Spectral fitting and IRIS-specific PpIX basis spectra

The hyperspectral images enable the application of spectral fitting for each image pixel. The linear least squares spectral fit process used herein, which has been reported on extensively^5,20,22^ with our LCTF-based imaging system, separates a normalized fluorescence spectrum into a weighted sum of its assumed constituent components (e.g., PpIX, its photoproducts, tissue autofluorescence, etc,), each of which is pre-defined by its known spectral shape that serves as a basis function during the fit. This process decouples the PpIX-specific signal from other fluorescence sources in the tissue. The spectral fitting algorithms used here run at 60 frames-per-second on a standard laptop, and thus are not a bottleneck for imaging time.

IRIS-specific PpIX basis spectra were established with experimental data collected from liquid PpIX phantoms with varying optical properties. Here, phosphate buffer saline (PBS) phantoms with blood volume fractions varying in the range of 1 to 3 [%], intralipid volume fractions in the range of 1 to 2 [%], and PpIX concentration in the range of 1 to 5 [µg/mL] were imaged. PBS used as the background medium also included 0.1% Tween 20, since the latter prevents PpIX aggregation which yields better fluorescence emission^24,43^. IRIS was mounted on the Zeiss Pentero operating microscope, and a box was fabricated to block ambient light, and ensure the only source of illumination came from the top (of the box), where an opening let the microscope illuminate the phantoms from a distance of approximately 25 cm. Liquid phantoms were poured into falcon tube (Corning, USA) caps 30 mm in diameter and 10 mm in depth, which were then placed into the box where they were illuminated with the Pentero’s blue light module (400 nm). An exposure time of 1 s was used to acquire the images, and data were averaged over the area of the phantom (disregarding pixels affected by specular reflection). These spectra were then averaged to account for the full range of phantom optical properties illuminated during an experiment.

### System sensitivity and comparison to sequential hyperspectral imaging using an LCTF

An extensive phantom study was completed to establish the linearity of response to PpIX and compare this performance to the LCTF system. Using averaged basis spectra, spectral fitting was performed on a series of liquid phantoms over a wide range of known PpIX concentrations. Liquid phantoms with 2[%] BVF, 1.5 [%] IVF, and PpIX concentrations of {0, 0.025, 0.05, 0.1, 0.2, 0.4, 0.8, 1.6, 3.2, 6.4} [µg/mL] were measured, and data were acquired with exposure times of 1 s (1 FPS), 0.25 s (4 FPS), and 0.167 s (6 FPS). To compare IRIS sensitivity to a more common temporal filtering system, the same set of phantoms was also imaged with a CMOS camera (PCO Edge) connected to an LCTF mounted to the microscope. Acquisition time of about 12 s, scanning 42 wavelengths, was necessary with the LCTF. For hyperspectral images acquired with IRIS, concentration estimates were generated using the averaged IRIS-specific PpIX spectra generated in Fig. 1. The averaged signal received from the 0 µg/mL phantom from this set served as an offset during the fitting process. For images acquired with the LCTF, fitting was performed using PpIX spectra from the literature, and a constant value of 1 as an offset. Once concentration estimates were computed, linear regression extracted the scaling factors needed to produce PpIX estimates that matched actual concentrations.

### Feasibility study in human glioma surgery

In a clinical case, IRIS and LCTF images of the same surgical field were compared. The subject was enrolled as part of a clinical research study (NCT02191488) approved by the Committee for Protection of Human Subjects at Dartmouth which serves as its Institutional Review Board, and provided informed consent to participate. Surgery began with IRIS mounted on the auxiliary port of the neurosurgical microscope. Once tumor was exposed, IRIS acquired 3 hyperspectral images of the visual field under white and blue light illumination. Exposure time was 167 ms (6 FPS) for these acquisitions, and all wavelengths were acquired simultaneously. RGB captures of the surgical field of view being displayed to the surgeon were taken under both blue and white illumination. Once complete, the surgical microscope was removed from the sterile field, undraped, the LCTF/PCO CMOS camera system was mounted to the same port, and the microscope was re-draped. The microscope was then moved back to the patient position, and a full set of hyperspectral images was acquired using the newly configured instrument under the same reflectance and fluorescence lighting conditions. In this configuration, we acquired 42 wavebands at 250 ms per waveband for a total acquisition time of 12 s. For all IRIS and LCTF recordings, RGB captures of the surgeon’s field of view were acquired for coregistration of the computed PpIX concentration maps. To generate quantitative concentration maps, spectral fitting was performed at each pixel of the hyperspectral stacks, using each system’s corresponding PpIX spectra and offsets as bases. The hyperspectral images acquired with both systems were also converted to RGB format. In order to compare IRIS and LCTF acquisitions, their respective images were coregistered by manually identifying 8 homologous feature point pairs evenly distributed within the cortical surface region to produce a similarity transformation which accounts for rotational, translational, and scaling differences between the two imaging systems. To refine the registration, Matlab’s OnePlusOneEvolutionary optimizer was applied using default settings. Once registered, the correlation coefficient between the two images was computed using the Matlab (corrcoef function). The respective concentration maps were also overlaid on images of the surgical field of view, which were presented to the surgeon. Notably, this registration process will not be necessary once the instrument is integrated onto the main port of the surgical microscope.

### Statistics and reproducibility

Mean values were used to convert spatially and spectrally distributed data into the graphical forms presented in Figs. 1, 2 and 3. For Fig. 1, an average of spatially distributed image pixel groups were formed and averaged for each spectrally distinct IRIS channel. In the case of Fig. 2, image pixel values in a central region of interest were averaged to generate the spectral response curve shown for all 64 IRIS channels which separated into the two 32-channel polarizations. Similarly, to generate source data in Fig. 3, pixels from images acquired from liquid phantoms with IRIS and LCTF instruments were averaged for each acquisition for different PpIX concentrations in phantoms of 2% blood volume fraction and 1.5% intralipid to replicate standard brain tissue optical properties. The process was repeated for different IRIS data acquisition rates and for both channel polarizations. Images in Fig. 4 were compared through correlation coefficient image processing.

Reproducibility was maintained by using standardized reference targets to estimate spatial resolution of IRIS and LCTF systems observed independently by 2 users. Similarly, commercial software and associated intrinsic functions (in Matlab) were used to compare image similarities between IRIS and LCTF results. Phantoms with known and repeatable optical properties served as gold standards for evaluation of quantitative PpIX concentrations obtained from IRIS and LCTF instruments. Use of multiple IRIS data acquisition rates and spectral data from both system polarizations which are recorded through distinct instrument data acquisition channels support reproducibility of results in Fig. 3, and again in Fig. 4. Comparisons between completely different imaging systems support reproducibility of results in Fig. 4, and their similarity with visually recorded fluorescence patterns also lend credibility to the findings. Data processed from two distinct polarization paths with IRIS demonstrate system reproducibility.

### Reporting summary

Further information on research design is available in the Nature Portfolio Reporting Summary linked to this article.

## Supplementary information

Supplementary Information

Description of Additional Supplementary Files

Supplementary Data 1

Supplementary Data 2

Supplementary Data 3

Reporting Summary

## Data Availability

Data in the form of fluorescence intensity, PpIX concentration values, and image pixel values are available on Figshare (10.6084/m9.figshare.22128842). Supplementary Data 1 and Supplementary Data 2 contain the source data for Figs. 2 and 3, respectively. Supplementary Data 3 contains test target images (raw 16-bit images along with scaled versions for visualization) used to determine spatial resolution of IRIS and LCTF systems (target images scaled for visualization also appear as Supplementary Figs. 1–2).

## Code availability

Scripts used to process data appearing in the paper can be found with corresponding data on Figshare (10.6084/m9.figshare.22128842) in the form of Matlab m files.
