# Supplementary material for: A birefringent spectral demultiplexer enables fast hyper-spectral imaging of protoporphyrin IX during neurosurgery
Source: Commun Biol. 2023 Mar 30;6:341. doi: 10.1038/s42003-023-04701-9 (PMC10060426; doi:10.1038/s42003-023-04701-9)
Supplement: Supplementary file 1 — Supplementary Information [file 42003_2023_4701_MOESM1_ESM.pdf]

Supplemental Figure 1
Title: Scaled Reference Target Image From LCTF System
Caption: Reference Target Image from LCTF system used to determine its spatial resolution. Image is compressed (windowed and leveled) for improved visualization.

Supplemental Figure 2
Title: Scaled Reference Target Image From IRIS System
Caption: Reference Target Image from IRIS system used to determine its spatial resolution. Image is compressed (windowed and leveled) for improved visualization.

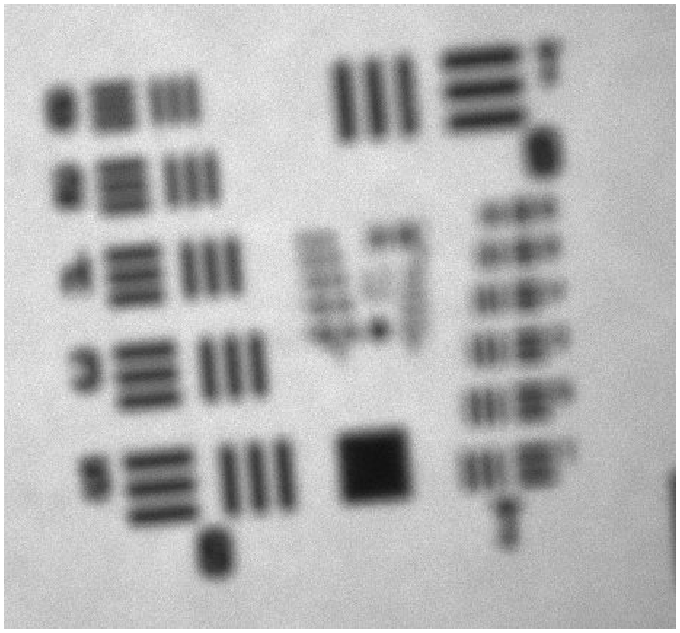

Supplemental Figure 1

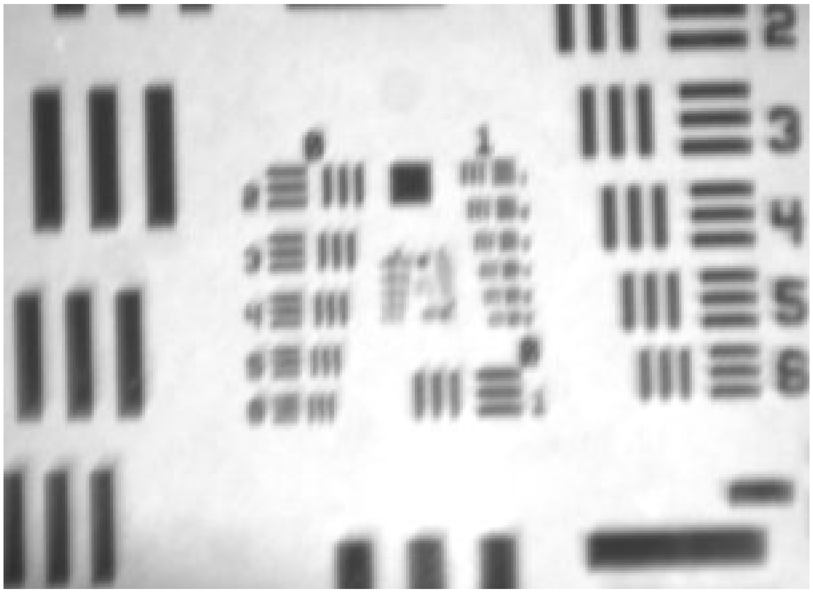

Supplemental Figure 2
