# Supplementary material for: A birefringent spectral demultiplexer enables fast hyper-spectral imaging of protoporphyrin IX during neurosurgery
Source: Commun Biol. 2023 Mar 30;6:341. doi: 10.1038/s42003-023-04701-9 (PMC10060426; doi:10.1038/s42003-023-04701-9)
Supplement: Supplementary file 2 — Description of Additional Supplementary Files [file 42003_2023_4701_MOESM2_ESM.pdf]

Description of Additional Supplementary Files

File Name: Supplementary Data 1
Description: The source data behind graph is Figure 2.

File Name: Supplementary Data 2
Description: The source data behind the graph in Figure 3.

File Name: Supplementary Data 3
Description: Zip folder of source image data of reference target for spatial resolution determinations of IRIS and LCTF imaging system including original 16-bit data and windowed versions for visualization for each imaging system.
