# Supplementary figures and images for: A birefringent spectral demultiplexer enables fast hyper-spectral imaging of protoporphyrin IX during neurosurgery
Source: Commun Biol. 2023 Mar 30;6:341. doi: 10.1038/s42003-023-04701-9 (PMC10060426; doi:10.1038/s42003-023-04701-9)

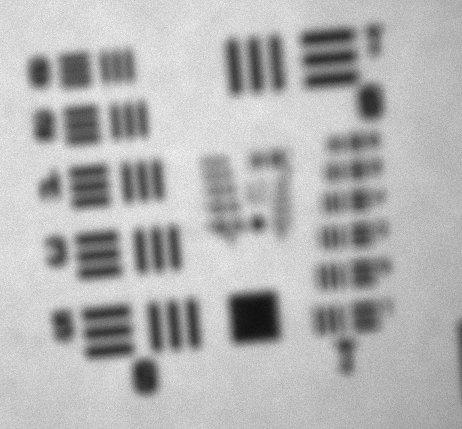

Supplement: Supplementary file 5 — Supplementary Data 3 [file 42003_2023_4701_MOESM5_ESM.zip › HIS_USAF.jpg]

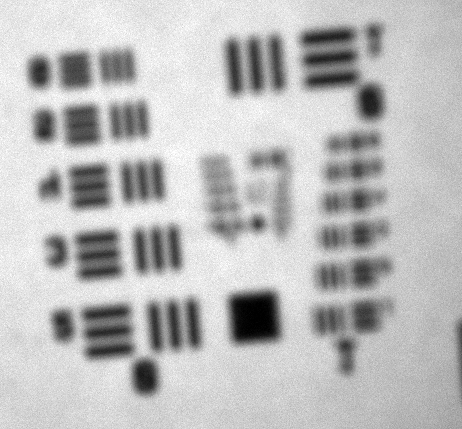

Supplement: Supplementary file 5 — Supplementary Data 3 [file 42003_2023_4701_MOESM5_ESM.zip › HIS_USAF.tif]

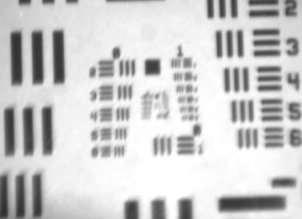

Supplement: Supplementary file 5 — Supplementary Data 3 [file 42003_2023_4701_MOESM5_ESM.zip › IRIS_USAF.jpg]

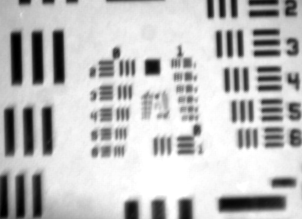

Supplement: Supplementary file 5 — Supplementary Data 3 [file 42003_2023_4701_MOESM5_ESM.zip › IRIS_USAF.tif]
